# High-Flow Nasal Oxygenation Is Associated with Improved Respiratory Stability During Moderate Multimodal Analgosedation for Pars Plana Vitrectomy: A Randomized Controlled Trial

**DOI:** 10.3390/jcm15207972

**Published:** 2026-10-15

**Authors:** Anita Vuković, Tomislav Jukić, Marija Štanfel, Krešimir Mandić, Slobodan Mihaljević, Dubravka Bartolek-Hamp

**Affiliations:** 1 Department of Anesthesiology, Reanimatology and Intensive Care Unit, General Hospital Dubrovnik, 20000 Dubrovnik, Croatia; anita_vukovic1@yahoo.com; 2 Clinic of Ophthalmology, University Hospital Centre Zagreb, 10000 Zagreb, Croatia; 3 Clinic of Anesthesiology, Reanimatology and Intensive Care Unit, University Hospital Centre Zagreb, 10000 Zagreb, Croatia

**Keywords:** high-flow nasal oxygenation, moderate sedation, spontaneous breathing, procedural sedation, pars plana vitrectomy, ASA physical status

## Abstract

**Background/Objectives:** High-flow nasal oxygenation (HFNO) may improve respiratory stability during procedural sedation, but treatment-associated differences across patient risk categories remain uncertain. We compared HFNO with low-flow nasal oxygenation (LFNO) during moderate multimodal analgosedation (MMAS) for pars plana vitrectomy (PPV) in patients with ASA physical status I–III. **Methods:** In this prospective, single-center randomized trial, 164 adults undergoing elective PPV under regional eye anesthesia with MMAS were randomized to HFNO (40 L·min^−1^, set FiO_2_ 0.40) or LFNO (5 L·min^−1^, intended to approximate a nominal FiO_2_ of 0.40). Sedation depth was monitored using the Patient State Index and evaluated using the Ramsay Sedation Scale. The primary outcome was maintenance of target oxygenation (SpO_2_ 94–98%) without hypoxemia (SpO_2_ < 90%), assessed by peripheral oxygen saturation (SpO_2_) at three predefined time points (T0, T1, and T2). Secondary outcomes included breathing frequency (BF), measured end-tidal CO_2_ (EtCO_2_), and clinically significant bradypnea (CSB), defined as ≥1 bradypnea episode (BF < 12 breaths·min^−1^ persisting ≥1 min) accompanied by SpO_2_ 90–93%, EtCO_2_ > 45 mmHg, or both. **Results:** The primary outcome showed no between-group difference. No hypoxemia (SpO_2_ < 90%) occurred. Oxygenation below the target range (SpO_2_ 90–93%) was less frequent with HFNO peri-procedurally (adjusted RR, 0.14; 95% CI, 0.03–0.56; *p* = 0.006) and post-procedurally (adjusted RR, 0.15; 95% CI, 0.04–0.64; *p* = 0.010). Significant treatment × ASA interactions were observed for BF and measured EtCO_2_ peri-procedurally. In exploratory ASA-stratified analyses, ASA III patients receiving HFNO had higher BF, fewer CSB episodes, and lower measured EtCO_2_ than those receiving LFNO. **Conclusions:** HFNO was associated with improved respiratory stability compared with LFNO, with treatment-associated differences varying across ASA strata.

## 1. Introduction

Moderate multimodal analgosedation (MMAS) combined with regional eye anesthesia (REA) is increasingly used during pars plana vitrectomy (PPV), providing effective analgesia, patient comfort, and appropriate surgical conditions while avoiding general anesthesia [1,2]. This approach is particularly advantageous in patients at increased anesthetic risk, including those with ASA physical status III, who may present additional perioperative challenges [3,4]. Consequently, maintaining respiratory safety during MMAS is an important component of modern vitreoretinal anesthesia [5].

Moderate analgosedation aims to preserve spontaneous breathing and cardiovascular stability without requiring airway intervention [6]. Nevertheless, sedative and opioid medications may cause respiratory depression, challenging the complex control of spontaneous breathing and the compensatory mechanisms involved in adapting ventilation to altered respiratory conditions [7]. Impaired upper-airway patency and hypoventilation may consequently increase the risk of oxygen desaturation, particularly in patients with cardiopulmonary disease, advanced age, or increased sensitivity to sedatives [8,9]. During PPV, airway interventions are especially undesirable because they may disrupt the sterile surgical field and interfere with delicate microsurgery. Moreover, individual responses to sedative and opioid medications are variable, making respiratory compromise difficult to predict despite standard monitoring [10]. Supplemental oxygen is therefore recommended during MMAS, although the optimal oxygenation strategy remains uncertain [11,12]. Because many contemporary oxygen-delivery devices may interfere directly with the vitreoretinal surgical field, oxygen supplementation during PPV is commonly provided through nasal cannulae using conventional low-flow nasal oxygenation (LFNO) or high-flow nasal oxygenation (HFNO) [2].

Although HFNO has been widely investigated for preventing peri-procedural hypoxemia, increasing evidence suggests that its physiological benefits extend beyond oxygen delivery [13,14]. These observations raise an important question: whether HFNO is associated with improved respiratory stability compared with LFNO during procedural sedation, and whether treatment-associated differences vary according to patient anesthetic risk [15,16,17].

Sawase et al. reported that nasal high flow with room air did not significantly reduce marked hypercapnia or time-weighted transcutaneous CO_2_ compared with low-flow supplemental oxygen during ERCP under moderate sedation; however, their primary outcome reached statistical significance when expressed as the risk difference, but not as the risk ratio [18].

Conversely, Horiuchi et al. cautioned that HFNO delivered with high inspired oxygen concentrations may maintain oxygen saturation despite respiratory depression, potentially creating a false impression of respiratory safety during procedural sedation [19].

We conducted a prospective randomized trial comparing HFNO with conventional LFNO in patients with ASA physical status I–III undergoing PPV under REA with MMAS. We hypothesized that HFNO would be associated with improved respiratory stability compared with LFNO and that treatment-associated differences would vary according to ASA physical status.

## 2. Materials and Methods

### 2.1. Study Design

This single-center, prospective, parallel-group, two-arm, stratified randomized controlled trial was prospectively registered at ClinicalTrials.gov (NCT04055077; 13 August 2019). The study protocol was approved by the Ethics Committee of the University Hospital Centre Zagreb (Institutional Approval No. 02/21 AG; Class: 8.1-19/188-3; 8 June 2020). Participant enrollment commenced only after institutional ethical approval had been obtained. Written informed consent was obtained from all participants before enrollment. The study was conducted at the University Hospital Centre Zagreb between June 2022 and September 2023. The trial was reported in accordance with the CONSORT 2025 Statement.

### 2.2. Participants

Eligible participants were adults (aged ≥18 years) scheduled for elective PPV under REA supplemented with MMAS. Patients with a body mass index (BMI) of 18.5–29.9 kg·m^−2^ and ASA physical status I–III were eligible for inclusion. Exclusion criteria were difficult airway, upper airway obstruction, BMI ≥ 30 kg·m^−2^, obstructive sleep apnea, chronic obstructive pulmonary disease, congenital heart defects, congestive heart failure, neuromuscular disease, ongoing chemotherapy or radiotherapy, anemia, hematologic or psychiatric disease, and known allergy to study medications or local anesthetics.

### 2.3. Randomization and Blinding

Eligible patients were screened during preanesthetic assessment by an independent anesthesiologist. Patients who provided written informed consent were enrolled in the study. Each participant was assigned a unique hospital patient admission number (UPHAN), which was used to conceal patient identity throughout data collection and analysis. Computer-generated randomization was performed using the GraphPad QuickCalcs Randomization Generator (https://www.graphpad.com/quickcalcs/randomize1.cfm; accessed before 1 June 2022). Three separate randomization sequences were generated, one for each ASA physical status stratum (ASA I, II, and III), using a 1:1 allocation ratio. Simple randomization rather than permuted-block randomization was used; therefore, no block size was specified, and exact numerical balance between treatment groups within each stratum was not guaranteed. Within each ASA stratum, treatment allocation was assigned sequentially according to subject number. Allocation concealment was ensured using sequentially numbered, opaque, sealed envelopes containing the treatment assignment and corresponding UPHAN. The envelopes were prepared by an administrator independent of the study protocol who was not involved in participant enrolment or treatment allocation. The attending anesthesiologist opened the envelope immediately before the procedure and implemented the assigned intervention. The attending anesthesiologist was aware of treatment allocation because the oxygen-delivery interfaces and flow rates were readily distinguishable. Bradypnea and CSB episodes were identified and documented prospectively during the procedure by the attending anesthesiologist using the predefined criteria. Data entry and statistical analysis were performed using coded treatment assignments without knowledge of treatment identity.

Objective perioperative data were recorded automatically by the anesthesia monitor (GE Healthcare Finland Oy, Helsinki, Finland) and documented in the anesthesia record under the participant’s UPHAN.

### 2.4. Interventions

Participants were randomly assigned to the intervention (HFNO) or control (LFNO) group within each ASA physical status stratum (ASA I–III). Participants assigned to HFNO received a heated, humidified oxygen–air mixture delivered at 40 L·min^−1^ via nasal cannula (Airvo™ 2, Fisher & Paykel Healthcare, Auckland, New Zealand).

A flow rate of 40 L·min^−1^ was used according to the predefined study protocol and was within the range of HFNO flow rates previously reported during adult procedural sedation [20]. Participants assigned to LFNO received oxygen at 5 L·min^−1^ via a standard nasal cannula. HFNO was delivered at a set FiO_2_ of 0.40, whereas LFNO at 5 L·min^−1^ was intended to approximate a nominal FiO_2_ of 0.40 according to commonly used estimates for LFNO [21]. However, the actual FiO_2_ delivered by LFNO was not measured and may have varied according to the patient’s ventilatory pattern and inspiratory flow [22].

Oxygenation was initiated immediately before induction of MMAS and continued for approximately 10 min after completion of PPV and discontinuation of MMAS.

### 2.5. Sedation Protocol

The MMAS protocol was standardized for all participants. Drug doses were calculated according to the patient’s ideal body weight. According to the Ophthalmology Department protocol, topical mydriatics (0.5% tropicamide, Tropicil Top Edol–Produtos Farmacêuticos, S.A., Linda-a-Velha, Portugal, and 10% phenylephrine, Phenylephrine Cooper, Cooper Pharmaceuticals, Greece) were administered before premedication. Oral benzodiazepine premedication was administered 45 min before MMAS and topical eye anesthesia (0.5% tetracaine, Tetrakain 0.5% eye drops, Gradska ljekarna Zagreb, Zagreb, Croatia). Patients aged <60 years received midazolam (Dormicum® 15 mg, Cheplapharm Arzneimittel GmbH, Greifswald, Germany) 0.1 mg·kg^−1^ (maximum dose, 7.5 mg), whereas those aged ≥60 years received diazepam (Normabel® 5 mg, Belupo d.d., Koprivnica, Croatia) 0.05 mg·kg^−1^ (maximum dose, 5.0 mg).

A crystalloid infusion (2 mL·kg^−1^·h^−1^) was established, and ondansetron (Ondansetron Kabi, 2 mg/mL, Fresenius Kabi d.o.o., Zagreb, Croatia) (4 mg) was administered intravenously. Remifentanil (Remifentanil Hameln, hameln pharma, Hameln, Germany) was infused at 0.05 µg·kg^−1^·min^−1^ for 5 min before regional eye block using an Agilia SP syringe infusion pump (Fresenius Vial, Brézins, France). A retrobulbar or sub-Tenon block was performed using 5–7 mL of a local anesthetic mixture comprising 2% lidocaine (Lidokain 2%, 100 mg/5 mL, B. Braun Adria d.o.o., Zagreb, Croatia) and 0.5% levobupivacaine (Fresenius Kabi, Brézins, France).in a 1:1 ratio. After the regional eye block, the remifentanil infusion was continued at an initial rate of 0.02 µg·kg^−1^·min^−1^ and subsequently titrated to maintain the protocol-defined Patient State Index (PSI) target of 80–85 during MMAS.

Sedation was additionally assessed clinically using the Ramsay Sedation Scale (RSS), a six-point scale: 1, anxious, agitated, or restless; 2, cooperative, oriented, and tranquil; 3, responds to commands only; 4, brisk response to a light glabellar tap or loud auditory stimulus; 5, sluggish response to a light glabellar tap or loud auditory stimulus; and 6, no response to stimulation [23]. Regional eye anesthesia provided the principal surgical analgesia, while remifentanil was used as an adjunctive component of MMAS to facilitate patient comfort and procedural tolerance. The remifentanil infusion was discontinued at the completion of PPV. Following discontinuation of MMAS, the assigned oxygenation strategy was continued for approximately 10 min. T2 was recorded immediately before discontinuation of LFNO or HFNO, while the assigned oxygenation strategy was still being delivered. Thereafter, patients breathed room air until achieving an Aldrete score of 9–10. Time to recovery was measured from discontinuation of oxygenation to achievement of this score.

Patients were monitored in the postanesthesia care unit (PACU) until an Aldrete score of 9–10 was achieved, after which they were discharged to the Department of Ophthalmology.

### 2.6. Monitoring and Measurements

#### 2.6.1. Monitoring

Hemodynamic (heart rate [HR] and mean arterial pressure [MAP]) and respiratory (peripheral oxygen saturation [SpO_2_] and breathing frequency [BF]) parameters were monitored using a multiparameter monitoring system integrated with the Datex-Ohmeda Aespire View^®^ anesthesia workstation (GE Healthcare, Chicago, IL, USA).

BF was measured by thoracic impedance derived from the ECG electrodes and was not calculated from the capnographic waveform. EtCO_2_ was measured using sidestream Microstream™ capnography with a Capnostream™ 35 Portable Respiratory Monitor (PM35MN; Oridion Medical 1987 Ltd., Jerusalem, Israel) and a Smart CapnoLine^®^ Plus O_2_ Adult/Intermediate oral/nasal CO_2_ sampling set. In the present study, the CO_2_ sampling port was positioned orally, close to the lower lip and separately from the nasal oxygen-delivery interface. The sampling flow rate was 50 mL·min^−1^. The surgical drape was secured proximally at the inferior margin of the surgical field, while its distal portion was elevated above the patient’s body, leaving an open gas-outlet space around the patient. Thoracic-impedance-derived BF was cross-checked against the capnographic waveform and capnographic respiratory-rate alarm, as well as the monitor’s apnoea/respiratory alarms. Because the surgical drapes precluded reliable direct observation of chest movement and airflow, clinical assessment included attempted verbal communication with the patient when respiratory abnormalities were suspected.

Sedation depth was assessed using the SedLine^®^ Brain Function Monitor integrated with the Root® monitoring system (Masimo Corporation, Irvine, CA, USA) [24]. PSI was used as an objective processed-EEG measure of the hypnotic component of sedation, while the Ramsay Sedation Scale (RSS) provided a complementary clinical assessment of sedation.

#### 2.6.2. Measurements

HR, SpO_2_, BF, measured EtCO_2_, and PSI were monitored continuously throughout the procedure. MAP was recorded at 5 min intervals. Monitoring began before initiation of oxygenation and continued throughout MMAS until 5 min after discontinuation of oxygenation. All measurements were documented in the anesthesia record together with sedative administration.

BF was routinely documented on the anesthesia chart at 5 min intervals. Any bradypnea episode was documented in real time at its occurrence, together with the contemporaneous SpO_2_ and measured EtCO_2_ values. Thus, episode-based bradypnea and CSB data reflected qualifying events occurring throughout the peri-procedural period, whereas T1 represented the predefined 15 min assessment.

The occurrence of the oculocardiac reflex (OCR) and cardiac arrhythmias was recorded throughout the procedure. PSI monitoring was initiated immediately before MMAS and continued throughout MMAS until 5 min after its completion.

Post-anesthesia recovery was assessed using the 10-point modified Aldrete score, comprising five components, each scored from 0 to 2 points, with higher scores indicating better recovery: activity (2 = moves all four extremities voluntarily or on command, 1 = moves two extremities, 0 = cannot move extremities), respiration (2 = breathes deeply and coughs freely, 1 = dyspnea or limited breathing, 0 = apnea), circulation (2 = blood pressure within ±20% of the preanesthetic level, 1 = deviation of 20–49%, 0 = or deviation of ≥50%), consciousness (2 = fully awake, 1 = arousable on calling, 0 = or unresponsive) and oxygen saturation (2 = maintains SpO_2_ > 92% on room air, 1 = requires supplemental oxygen to maintain SpO_2_ > 90%, 0 = SpO_2_ ≤ 90% with O_2_ supplementation) [25].

#### 2.6.3. Time Points

The predefined study time points were: T0 (pre-procedural), after premedication and before initiation of the assigned oxygenation strategy and MMAS; T1 (peri-procedural), 15 min after initiation of oxygenation, and MMAS when a steady state of MMAS had been achieved; and T2 (post-procedural), immediately before discontinuation of LFNO or HFNO, while the assigned oxygenation strategy was still being delivered.

### 2.7. Outcomes

#### 2.7.1. Primary Outcome

The prespecified primary outcome was maintenance of oxygenation above the level of hypoxemia, assessed by peripheral oxygen saturation (SpO_2_) at three predefined time points: T0 (pre-procedural), T1 (peri-procedural), and T2 (post-procedural). In accordance with the prospectively registered outcome definition, target oxygenation was defined as SpO_2_ 94–98%, consistent with the British Thoracic Society guideline recommendations [11]. For study purposes, SpO_2_ 90–93% was classified as oxygenation below the target range, SpO_2_ > 98% as oxygenation above the target range, and SpO_2_ < 90% as hypoxemia.

#### 2.7.2. Secondary Outcomes

Secondary outcomes included EtCO_2_, BF, bradypnea (BP), clinically significant bradypnea (CSB), MAP, HR, and sedation depth assessed by PSI.

#### 2.7.3. Outcome Definitions

Target physiological ranges and predefined intervention thresholds were established prospectively. For the purposes of this study, respiratory stability during MMAS was operationally defined as maintenance of spontaneous breathing with target oxygenation (SpO_2_ 94–98%), normopnea (BF within the predefined range of 12–20 breaths·min^−1^), and EtCO_2_ within the predefined range of 35–45 mmHg.

The target sedation level corresponded to a Patient State Index (PSI) of 80–85. Sedation deeper than the target range (PSI < 80) and oxygenation below the target range (SpO_2_ < 94%) were recorded as study outcomes. Predefined safety thresholds requiring immediate clinical intervention were deep sedation (PSI < 70) and hypoxemia (SpO_2_ < 90%).

Target oxygenation was determined according to the British Thoracic Society guideline for oxygen therapy in adults without chronic obstructive pulmonary disease [11].

Oxygenation below the target range was defined as SpO_2_ 90–93%, whereas oxygenation above the target range was defined as SpO_2_ > 98%.

Bradypnea was defined as BF <12 breaths·min^−1^ persisting for at least 1 min. CSB was defined as at least one qualifying bradypnea episode occurring concomitantly with EtCO_2_ > 45 mmHg, oxygenation below the predefined target range (SpO_2_ 90–93%), or both. BF and EtCO_2_ were monitored continuously throughout the peri-procedural period, and qualifying episodes were identified and documented during the procedure. Transient changes not meeting the duration and co-occurrence criteria above were not classified as CSB.

Hypotension and bradycardia were defined as decreases >15% from baseline, whereas hypertension and tachycardia were defined as increases >15% from baseline. New-onset arrhythmia, defined as three consecutive ectopic beats recorded within 15 s, was classified as procedure-dependent. A sudden decrease in HR ≥ 20% related to surgical pressure on eye structures was accepted as an oculocardiac reflex (OCR).

### 2.8. Adverse Events

Adverse events were classified as sedation-related, respiratory, hemodynamic, or PPV procedure-related events occurring during MMAS. Sedation-related adverse events included deeper-than-target sedation (PSI < 80). Respiratory adverse events included hypoventilation, apnea, hypoxemia, airway obstruction, and clinically significant bradypnea. Hemodynamic adverse events included hypotension, hypertension, bradycardia, tachycardia, and new-onset arrhythmia. PPV procedure-related adverse events included the oculocardiac reflex, incomplete REA, nausea, patient discomfort related to surgical draping, and restlessness. All adverse events were managed according to institutional clinical practice to maintain patient safety and appropriate surgical conditions.

### 2.9. Protocol Deviations

Major protocol deviations were defined as events requiring discontinuation of protocol-compliant study procedures because safe continuation of the assigned intervention and/or surgical procedure was not possible. Temporary interruption of surgery alone was not considered a protocol deviation when the assigned oxygenation strategy and protocol-compliant monitoring could subsequently be resumed and completed.

### 2.10. Statistical Analysis

The sample-size calculation was based on preliminary data from a pre-protocol pilot analysis performed in 2019, which provided anticipated mean SpO_2_ values of 98% versus 92% and a common standard deviation of 6.5 percentage points.

Using these assumptions, a two-independent-sample continuous-endpoint calculation with a two-sided α of 0.05, 80% power, and a 1:1 allocation ratio indicated that a minimum of 20 participants per group was required. The below-target SpO_2_ category (90–93%) was not the endpoint used for the sample-size calculation and was not separately powered. It was analyzed as a secondary categorical oxygenation outcome.

The calculated sample size represented the minimum required enrollment rather than a fixed maximum enrollment target. The enrollment minimum was applied within each ASA stratum, and recruitment continued until a minimum of 21 participants per treatment group was achieved in the slowest-enrolling ASA stratum. Because recruitment in the other ASA strata continued concurrently until this stopping criterion was reached, the final number of randomized participants exceeded the calculated minimum.

Data distribution was assessed using the Shapiro–Wilk test. The primary and secondary outcomes were analyzed in the predefined per-protocol population, comprising randomized participants who completed protocol-compliant study procedures without major protocol deviations and with the required protocol data available. Continuous variables were compared using the unpaired Student’s *t* test or one-way analysis of variance (ANOVA) with Tukey’s honestly significant difference (HSD) post hoc test, as appropriate. Categorical variables were compared using the χ^2^ test or Fisher’s exact test, as appropriate. Continuous outcomes are reported as the mean ± standard deviation (SD), together with the between-group difference, 95% confidence interval (CI), and *p*-value, as appropriate. Binary outcomes are reported as relative risks (RRs) with corresponding 95% CIs and *p* values. For binary respiratory outcomes, overall treatment effects adjusted for ASA physical status were estimated using modified Poisson regression with robust standard errors, with treatment group and ASA physical-status class included as covariates. Adjusted RRs with 95% CIs were reported. The categorical distribution of SpO_2_ across predefined ranges (>98%, 94–98%, and <94%) at each study time point was additionally summarized using forest plots of RRs with 95% CIs. For comparisons containing a zero-event cell, a continuity correction of 0.5 was applied to all cells of the corresponding 2 × 2 table for calculation of the RR and 95% CI.

For repeated BF and measured EtCO_2_ measurements, post-baseline treatment effects were additionally assessed using baseline-adjusted linear regression, with the T1 or T2 value as the dependent variable, treatment group as the main factor, the corresponding T0 value as a covariate, and ASA physical-status class as an adjustment factor. Adjusted mean differences with 95% CIs were reported. Treatment × ASA interactions for BF and measured EtCO_2_ at T1 were assessed using two-factor linear models including treatment group, ASA physical-status class, and their interaction.

ASA-stratified analyses were prespecified as part of the stratified trial design. Because no multiplicity-adjusted confirmatory testing hierarchy was prespecified for the individual stratum-specific and time-specific comparisons, these estimates were interpreted cautiously and were not considered independently confirmatory. Sensitivity analyses included all randomized participants using available outcome data up to the point of protocol deviation. For ASA III, additional extreme-case analyses were performed for CSB and below-target SpO_2_ at T1 and T2 by alternately assigning the event to the excluded LFNO or HFNO participant. Within each ASA physical-status stratum, LFNO and HFNO continuous outcomes at individual assessment time points were compared using two-sided independent-samples Student’s *t*-tests. Exploratory within-group comparisons of BF between T0, T1, and T2 in ASA III patients receiving HFNO were performed using two-sided paired Student’s *t*-tests, with 95% confidence intervals calculated for the mean paired differences. Statistical analyses were performed using SAS software, version 9.4 (SAS Institute Inc., Cary, NC, USA).

## 3. Results

### 3.1. Participants Flow and Study Population

During the study, 176 patients were assessed for eligibility. Seven patients were excluded before randomization (five refused regional eye anesthesia and two declined to participate). At the time the enrolment stopping criterion was reached, 169 patients had been randomized and stratified according to ASA physical status. Eighty-eight patients were allocated to LFNO and 81 to HFNO. Five randomized patients were not included in the final per-protocol analysis: two because of incomplete data collection (HFNO, ASA I, *n* = 1; LFNO, ASA II, *n* = 1) and three because of major protocol deviations: HFNO-related moisture in the surgical field (HFNO, ASA II, *n* = 1), excessive sedation (LFNO, ASA III, *n* = 1), and peri-procedural restlessness incompatible with safe continuation of ophthalmic surgery (HFNO, ASA III, *n* = 1). A sensitivity analysis was additionally performed using available outcome data from randomized participants excluded from the per-protocol population, including data recorded up to the point of protocol deviation.

**Figure 1:**
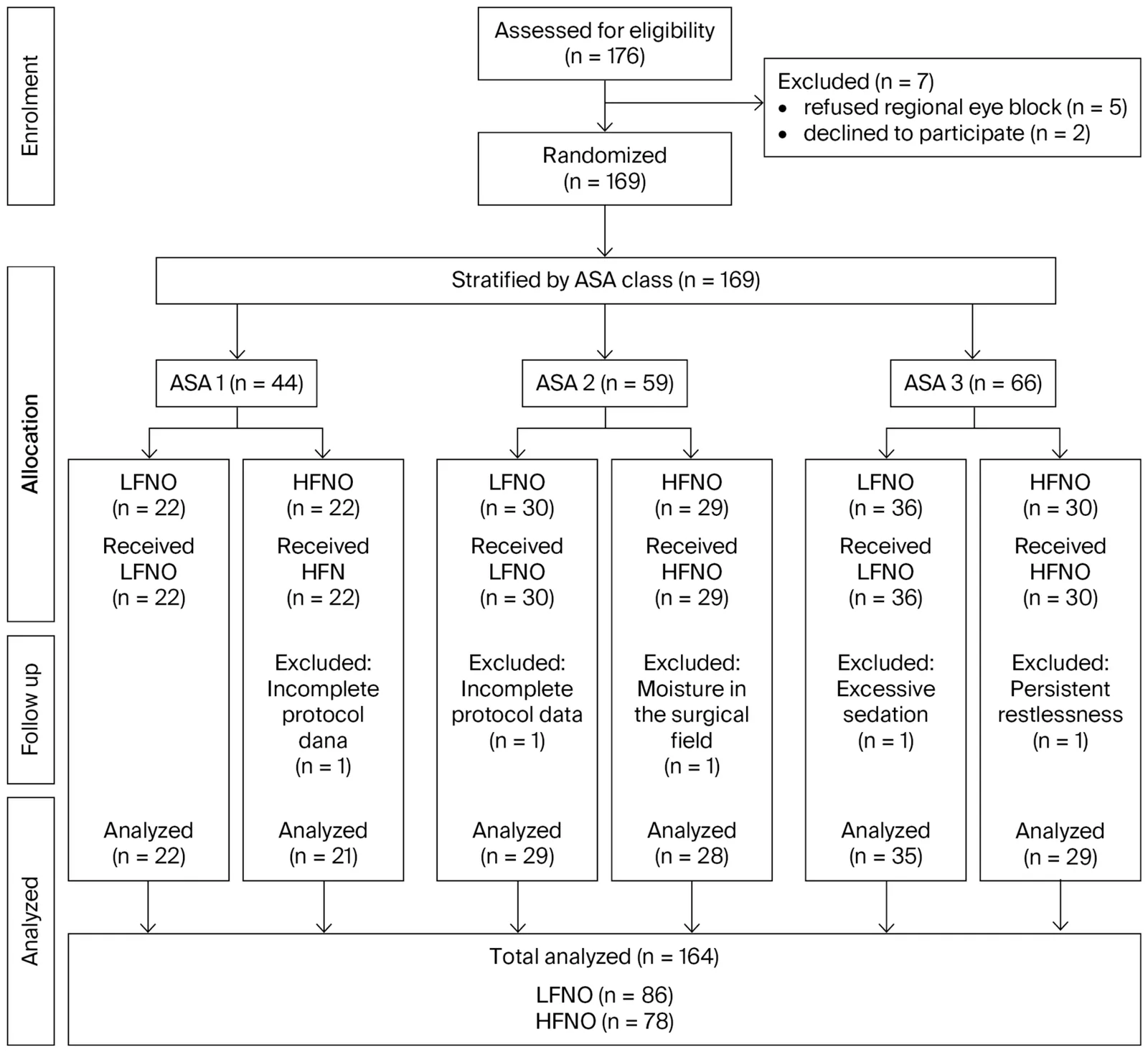
CONSORT flow diagram of participant enrollment, randomization, allocation, follow-up, and analysis. LFNO, low-flow nasal oxygenation; HFNO, high-flow nasal oxygenation.

Data from 164 patients were included in the final per-protocol analysis: 86 in the LFNO group (ASA I, *n* = 22; ASA II, *n* = 29; ASA III, *n* = 35) and 78 in the HFNO group (ASA I, *n* = 21; ASA II, *n* = 28; ASA III, *n* = 29) (Figure 1).

### 3.2. Baseline Demographic and Procedural Characteristics

The study groups were comparable according to demographic and procedural characteristics related to PPV and MMAS (Table 1). All analyzed patients achieved an Aldrete score of 9–10 before discharge from the PACU, with a mean time of approximately 5–6 min across ASA strata (Table 1).

### 3.3. Primary Outcome Results

No participant in either treatment group developed hypoxemia (SpO_2_ < 90%) at any of the predefined assessment time points; therefore, the primary outcome showed no between-group difference. Mean SpO_2_ was 97 ± 1.8% with LFNO and 96 ± 2.7% with HFNO at T0 (*p* = 0.106), 97 ± 2.6% and 98 ± 2.0%, respectively, at T1 (*p* = 0.015), and 98 ± 1.5% and 98 ± 1.7%, respectively, at T2 (*p* = 0.041).

**Figure 2:**
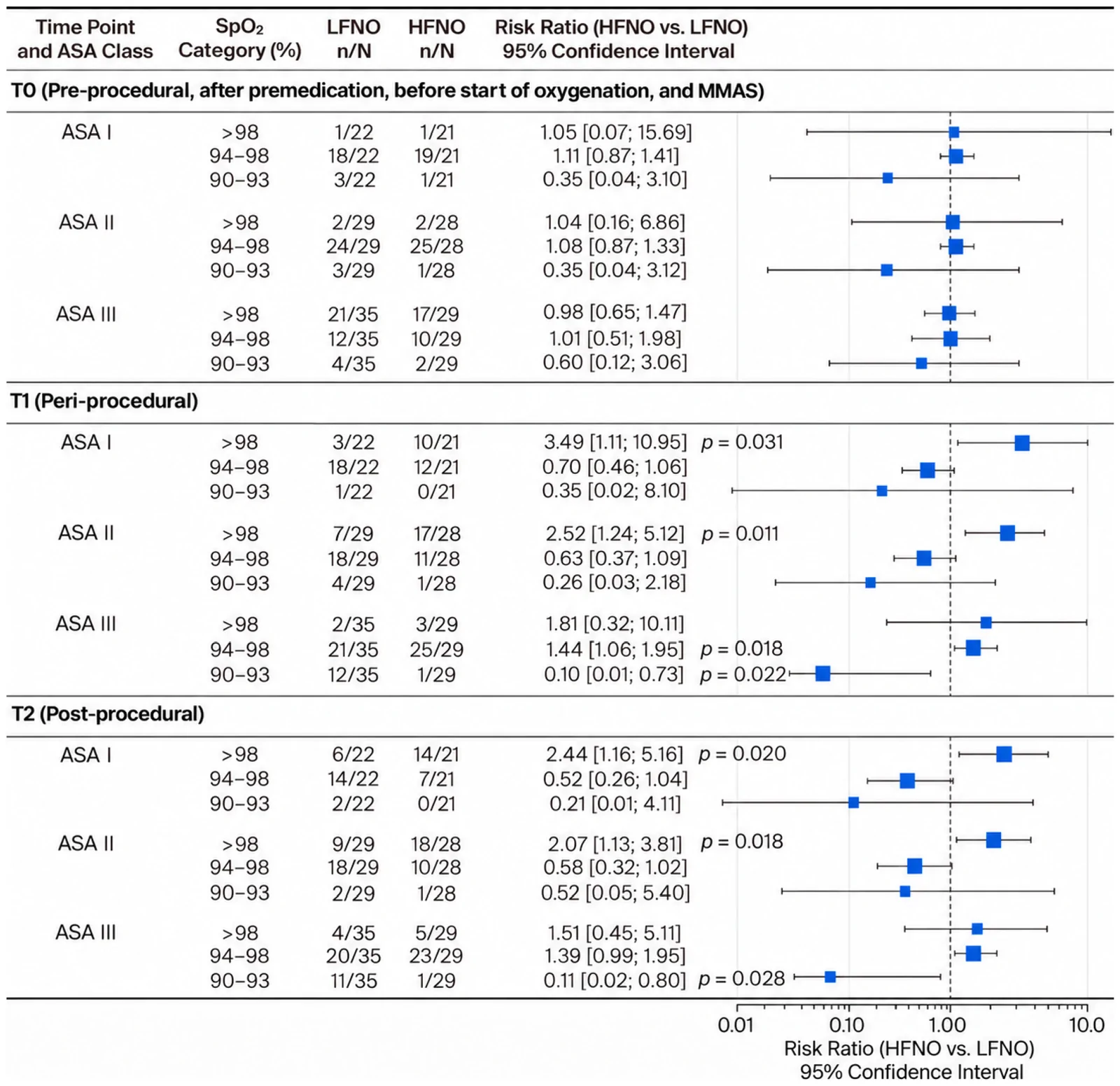
SpO_2_ categories across predefined assessment time points according to ASA physical-status class and oxygenation strategy. Event counts (*n*/N) and risk ratios (RRs) with 95% confidence intervals are shown for LFNO and HFNO. T0 represents the pre-procedural assessment after premedication and before initiation of the assigned oxygenation strategy and MMAS and is presented descriptively; T1 and T2 represent the peri-procedural and post-procedural assessments, respectively. SpO_2_ 94–98% was the predefined target range, SpO_2_ 90–93% represented oxygenation below the target range, and SpO_2_ > 98% represented oxygenation above the target range. No SpO_2_ < 90% occurred. ASA-stratified category-specific RRs are exploratory. ASA, American Society of Anesthesiologists; HFNO, high-flow nasal oxygenation; LFNO, low-flow nasal oxygenation; MMAS, moderate multimodal analgosedation; RR, risk ratio; SpO_2_, peripheral oxygen saturation.

Under the protocol-defined oxygen-delivery settings, oxygenation remained within the target range (SpO_2_ 94–98%) at T1 in 57/86 (66.3%) patients receiving LFNO and 48/78 (61.5%) patients receiving HFNO. In the ASA-adjusted analysis, HFNO was associated with a lower risk of oxygenation below the target range (SpO_2_ 90–93%) at T1 (adjusted RR, 0.14; 95% CI, 0.03–0.56; *p* = 0.006) and T2 (adjusted RR, 0.15; 95% CI, 0.04–0.64; *p* = 0.010). In the exploratory ASA-stratified analyses, HFNO was associated with a higher proportion of oxygenation above the target range (SpO_2_ > 98%) at T1 in ASA I (RR, 3.49; 95% CI, 1.11–10.95; *p* = 0.032) and ASA II patients (RR, 2.52; 95% CI, 1.24–5.12; *p* = 0.011) (Figure 2).

### 3.4. Secondary Outcomes Results

At T1, BF was numerically lower with HFNO than with LFNO in ASA I (12 vs. 14 breaths·min^−1^) and ASA II (11 vs. 13 breaths·min^−1^), whereas the direction was reversed in ASA III (16 vs. 10 breaths·min^−1^). A significant treatment × ASA interaction was observed at T1 (F(2,158) = 31.44; *p* < 0.0001), indicating that the treatment-associated difference in BF varied across ASA strata. In ASA III, BF decreased from T0 to T1 with LFNO (14 ± 3.6 to 10 ± 3.2 breaths·min^−1^), whereas it increased with HFNO (14 ± 2.8 to 16 ± 1.9 breaths·min^−1^). The overall baseline-adjusted between-group estimate at T1 was 1.34 breaths·min^−1^ (95% CI, 0.35–2.34; *p* = 0.008), and at T2 was −1.15 breaths·min^−1^ (95% CI, −1.95 to −0.35; *p* = 0.005); however, these pooled estimates should be interpreted in the context of the treatment × ASA interaction. In ASA III patients receiving HFNO (*n* = 29), exploratory paired comparisons showed a significant increase in BF from T0 to T1 (mean difference, +1.97 breaths/min; 95% CI, 0.97–2.96; *p* < 0.001), followed by a significant decrease from T1 to T2 (−3.10 breaths/min; 95% CI, −4.35 to −1.86; *p* < 0.001), with no significant difference between T0 and T2 (*p* = 0.115).

At T1, measured EtCO_2_ was similar between HFNO and LFNO in ASA I (34 vs. 35 mmHg) and ASA II (33 vs. 35 mmHg), whereas a larger between-group difference was observed in ASA III (40 vs. 47 mmHg). In ASA III, measured EtCO_2_ increased from T0 to T1 with LFNO (38 ± 3.3 to 47 ± 2.1 mmHg), whereas it decreased with HFNO (44 ± 3.3 to 40 ± 3.9 mmHg). A significant treatment × ASA interaction was observed at T1 (F(2,158) = 7.81; *p* = 0.0006), indicating that the treatment-associated difference in measured EtCO_2_ varied across ASA strata.

**Figure 3:**
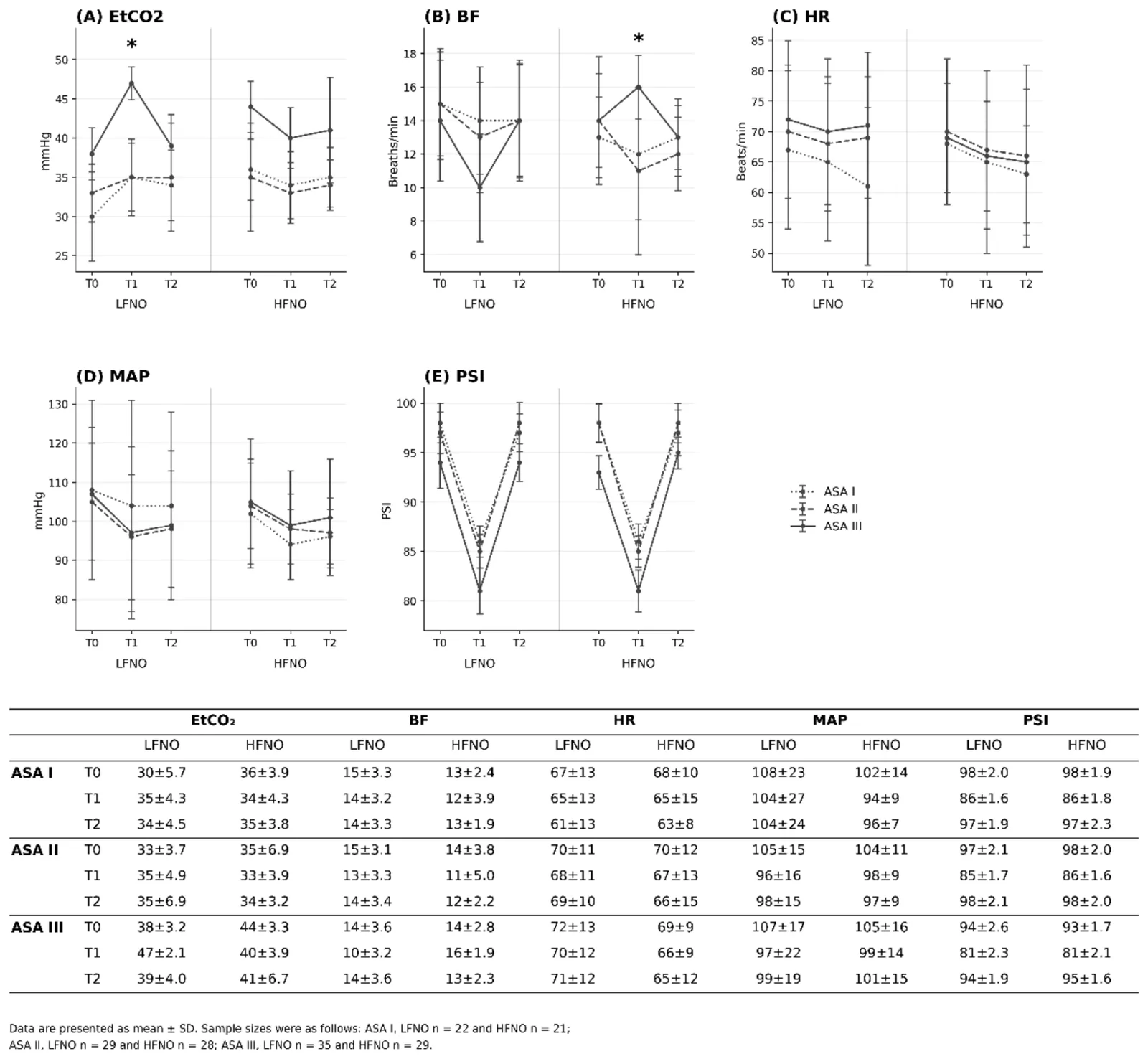
Secondary outcomes across procedural time points in ASA I–III patients receiving HFNO or LFNO during MMAS. Panels show changes in (**A**) end-tidal carbon dioxide (EtCO_2_), (**B**) breathing frequency (BF), (**C**) heart rate (HR), (**D**) mean arterial pressure (MAP), and (**E**) Patient State Index (PSI) at pre-procedural (T0), peri-procedural (T1), and post-procedural (T2) time points. Different line styles represent ASA physical-status classes I, II, and III, as indicated in the figure legend. Data are presented as mean ± SD (vertical error bars). * *p* < 0.05 for the between-group LFNO-versus-HFNO comparison within the corresponding ASA physical-status stratum and assessment time point (two-sided independent-samples Student’s *t*-test). ASA, American Society of Anesthesiologists physical status; LFNO, low-flow nasal oxygenation; HFNO, high-flow nasal oxygenation; MMAS, moderate multimodal analgosedation.

Peri-procedural MAP and HR remained within the predefined safety limits (±15% from baseline), while mean PSI values remained within the target sedation range across ASA groups. No significant differences in MAP, HR, or PSI were observed between HFNO and LFNO (Figure 3).

PSI values at T0, T1, and T2 according to oxygenation strategy and ASA physical status are shown in Figure 3E and reported numerically in Table 2, together with the corresponding clinically recorded RSS distributions. During MMAS, recorded RSS scores were limited to 2 and 3 in both treatment groups. The observed PSI values and RSS distributions were similar between LFNO and HFNO within each ASA stratum (Table 2).

### 3.5. Bradypnea and Clinically Significant Bradypnea

**Figure 4:**
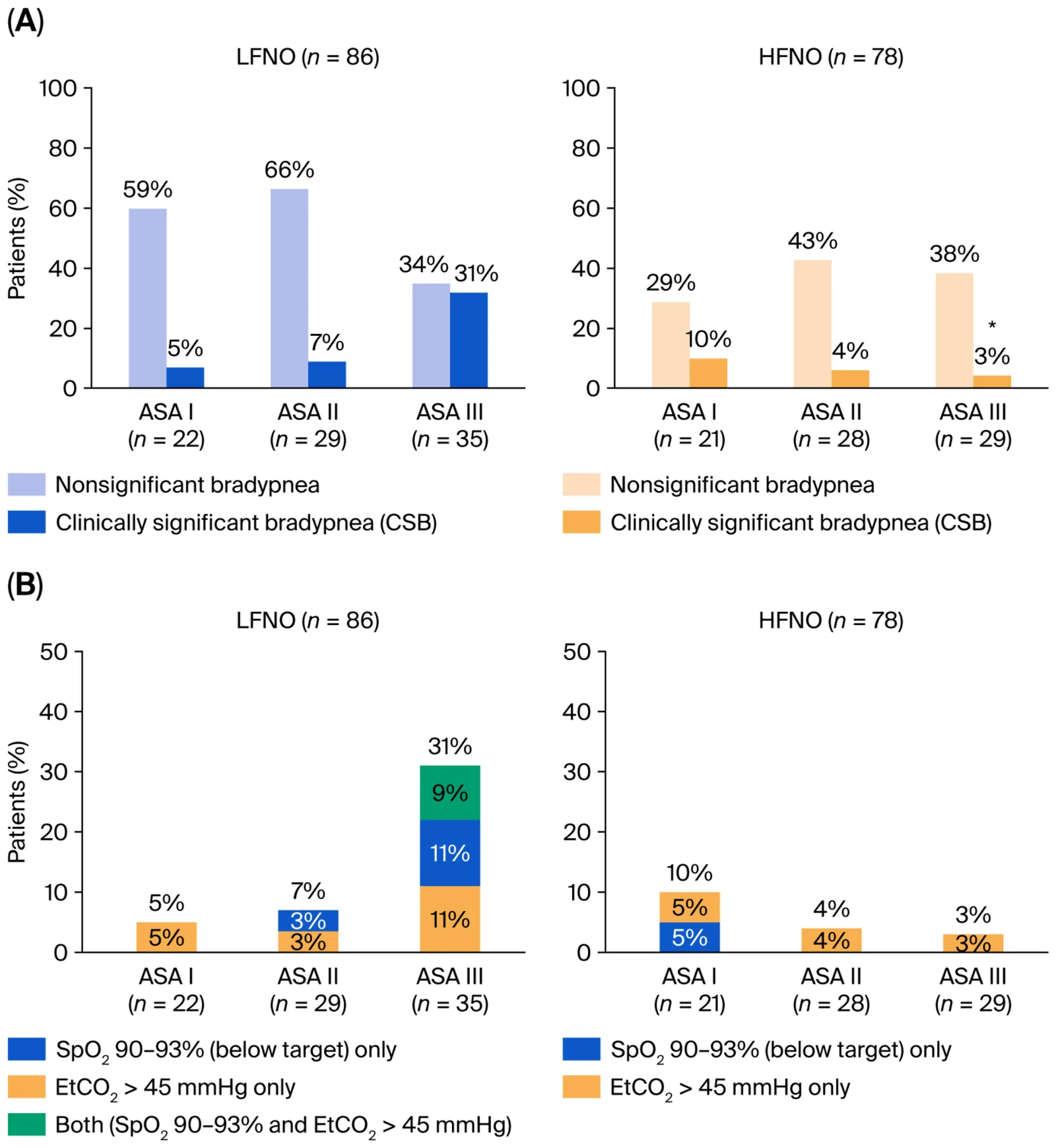
Bradypnea and clinically significant bradypnea (CSB) during MMAS in ASA I–III patients receiving LFNO or HFNO. Bradypnea was defined as breathing frequency <12 breaths·min^−1^ persisting for at least 1 min. CSB was defined as ≥1 qualifying bradypnea episode occurring with measured EtCO_2_ > 45 mmHg, oxygenation below the target range (SpO_2_ 90–93%), or both. Episode data were based on real-time anesthesia-chart documentation during continuous monitoring and therefore represent events occurring throughout the peri-procedural period rather than measurements restricted to T1. (**A**) shows the occurrence of bradypnea and CSB across ASA I–III groups. In ASA III patients, CSB occurred less frequently with HFNO than with LFNO (3% vs. 31%; *p* = 0.008). (**B**) shows the components accompanying CSB: measured EtCO_2_ > 45 mmHg, oxygenation below the target range (SpO_2_ 90–93%), or both. Among ASA III patients, CSB accompanied by measured EtCO_2_ > 45 mmHg, below-target oxygenation, or both was less frequent with HFNO than with LFNO. Percentages are rounded to the nearest whole number; therefore, displayed component percentages may not sum exactly to the total. * indicates a significant difference in CSB between LFNO and HFNO in ASA III patients (*p* = 0.008). ASA, American Society of Anesthesiologists physical status; CSB, clinically significant bradypnea; EtCO_2_, end-tidal carbon dioxide; HFNO, high-flow nasal oxygenation; LFNO, low-flow nasal oxygenation; MMAS, moderate multimodal analgosedation; SpO_2_, peripheral oxygen saturation.

At least one peri-procedural bradypnea episode occurred less frequently with HFNO than with LFNO (42% vs. 67%; RR, 0.63; 95% CI, 0.47–0.84; *p* = 0.0016). In the ASA-adjusted analysis, HFNO was associated with a lower risk of peri-procedural bradypnea (adjusted RR, 0.65; 95% CI, 0.48–0.88; *p* = 0.005) and CSB (adjusted RR, 0.33; 95% CI, 0.11–0.94; *p* = 0.039). CSB occurred in 2/21 (10%), 1/28 (4%), and 1/29 (3%) of ASA I, II, and III patients receiving HFNO, respectively, and in 1/22 (5%), 2/29 (7%), and 11/35 (31%) of those receiving LFNO, respectively. Among ASA III patients, CSB involving measured EtCO_2_ > 45 mmHg, below-target oxygenation, or both occurred less frequently with HFNO than with LFNO (Figure 4).

Sensitivity analyses including all randomized participants with available data up to the point of protocol deviation were consistent with the per-protocol findings. Within ASA III, the direction of the CSB and below-target SpO_2_ findings was unchanged after inclusion of the excluded participants and remained robust in extreme-case analyses.

### 3.6. Adverse Events During MMAS

Deeper-than-target sedation was uncommon overall, with events occurring predominantly among ASA III patients (Table 3). Among 4/35 (11%) ASA III patients receiving LFNO who developed deeper-than-target sedation, all four experienced clinically significant bradypnea (4/4) and oxygenation below the target range (SpO_2_ 90–93%; 4/4). Given the small number of events, these findings are reported descriptively and should be interpreted cautiously. Other adverse events were infrequent, with no significant differences between LFNO and HFNO within any ASA physical status stratum (Table 3).

## 4. Discussion

This randomized trial demonstrated that, under the protocol-defined oxygen-delivery settings and protocol-guided sedation during MMAS for PPV, no hypoxemia (SpO_2_ < 90%) occurred. HFNO was associated with higher BF and fewer CSB episodes, most prominently in patients with ASA physical status III. In these patients, HFNO was associated with higher peri-procedural BF, lower measured EtCO_2_, fewer episodes of bradypnea, and a significantly lower occurrence of CSB than LFNO, while also reducing the occurrence of oxygenation below the predefined target range (SpO_2_ < 94%) during the peri-procedural and recovery periods. In contrast, patients with ASA physical status I–II maintained satisfactory respiratory stability with both LFNO and HFNO, although HFNO more frequently resulted in SpO_2_ > 98%.

In the prespecified ASA-stratified analyses, the most pronounced treatment-associated differences were observed in ASA III patients. Patients with ASA physical status III appeared more vulnerable to respiratory compromise during MMAS, despite receiving lower remifentanil infusion rates than ASA I–II patients. This pattern may reflect greater sensitivity to the respiratory depressant effects of analgosedation in this subgroup, although individual sensitivity to remifentanil was not directly assessed. In ASA III patients, HFNO was associated with higher BF, lower measured EtCO_2_, and fewer CSB episodes than LFNO. Although opioid-based analgosedation is associated with respiratory depression, previous physiological research during propofol sedation has reported a reduction in respiratory rate during nasal high-flow delivery [26]. The contrasting BF pattern observed in ASA III patients receiving HFNO may have several explanations, including stimulation or arousal associated with high-flow delivery, baseline differences between treatment groups, or measurement effects related to thoracic-impedance-derived BF. Furthermore, lower measured EtCO_2_ during HFNO cannot necessarily be interpreted as improved ventilation because oral side stream sampling may be influenced by high nasal flow. Thus, although the observed associations suggest more favorable respiratory outcomes with HFNO in ASA III patients under the study conditions, they do not establish improved ventilatory efficiency, preservation of spontaneous ventilation, or a specific physiological mechanism. In the present study, in ASA III patients receiving HFNO, BF increased significantly from baseline (T0) to the peri-procedural assessment (T1), followed by a significant decrease at recovery (T2).

Previous studies have predominantly attributed the benefits of HFNO during procedural sedation to improved oxygenation and reduced hypoxemia [27,28,29]. In contrast, our findings suggest that the differences associated with HFNO may extend beyond oxygenation alone. Sawase et al. found no significant difference in time-weighted transcutaneous CO_2_ and no statistically significant reduction in marked hypercapnia with nasal high flow compared with low-flow supplemental oxygen during procedural sedation [18]. This finding does not establish a direct ventilatory-support effect of high-flow therapy. This interpretation is consistent with evidence that supplemental oxygen, particularly at higher inspired oxygen fractions, may preserve SpO_2_ despite evolving hypoventilation [19,30].

Among patients with ASA physical status III, deeper-than-target sedation occurred more frequently despite lower remifentanil infusion rates than in ASA I–II patients. Furthermore, in ASA III patients receiving LFNO, deeper-than-target sedation was associated with CSB and below-target oxygenation, reinforcing the apparent respiratory vulnerability of this subgroup. Other adverse events were infrequent and did not differ significantly between LFNO and HFNO within ASA strata. Advanced age, higher ASA physical status, and underlying cardiopulmonary disease have been associated with an increased risk of respiratory compromise during procedural sedation, and HFNO has been investigated as an oxygenation strategy in higher-risk populations [8,31,32,33]. Our findings extend these observations by suggesting that respiratory outcomes associated with HFNO may be particularly relevant to patients with ASA physical status III undergoing PPV under REA and MMAS.

In contrast to its more pronounced effects on respiratory stability, HFNO produced relatively limited differences in oxygenation when HFNO was delivered at a set FiO_2_ of 0.40 and LFNO at a nominal FiO_2_ of 0.40. Oxygen saturation remained within the predefined target range (SpO_2_ 94–98%) in most patients, and no hypoxemia (SpO_2_ < 90%) occurred in either group, indicating that LFNO was generally sufficient to maintain oxygenation during MMAS. Nevertheless, preserved SpO_2_ did not necessarily indicate preserved ventilatory stability, particularly in patients with ASA physical status III, in whom HFNO was associated with higher BF, lower measured EtCO_2_, and less CSB than LFNO. Although significant treatment × ASA interactions were observed for BF and measured EtCO_2_ at T1, the prespecified ASA-stratified findings should be interpreted cautiously because of the relatively small stratum-specific sample sizes and the absence of a multiplicity-adjusted confirmatory testing hierarchy; ASA class itself was not a randomized exposure. These findings highlight the distinction between maintaining oxygenation and monitoring respiratory function during procedural sedation. Previous studies have frequently reported lower rates of hypoxemia with HFNO; however, these investigations commonly compared oxygen-delivery strategies differing in flow and/or FiO_2_ [34,35,36]. By using HFNO at a set FiO_2_ of 0.40 and LFNO at a nominal FiO_2_ of 0.40, our study sought to limit differences in oxygen concentration and to compare respiratory outcomes between HFNO and LFNO under these protocol-defined settings. The more frequent occurrence of SpO_2_ > 98% among ASA I–II patients receiving HFNO further suggests that oxygen therapy should be individualized according to patient characteristics and clinical requirements.

Accurate monitoring remains fundamental to respiratory safety during MMAS, particularly in patients at increased anesthetic risk. Although pulse oximetry reliably detects hypoxemia, SpO_2_ may remain within the target range despite evolving hypoventilation when supplemental oxygen is administered [37,38]. Continuous assessment of BF in the present study, together with objective sedation monitoring, facilitated detection of respiratory compromise that might not have been apparent from pulse oximetry alone.

EtCO_2_ monitoring provided additional information on respiratory status, although the measured values may have been influenced by HFNO delivery. These findings support integrated monitoring of oxygenation, ventilation, and sedation for the timely detection of respiratory compromise during MMAS [1,37,38].

Several limitations should be considered when interpreting these findings. First, this was a single-center study with a relatively small number of patients with ASA physical status III, and the study was not powered for ASA-stratified subgroup comparisons or individual CSB components; these analyses should therefore be considered exploratory. Second, the outcomes were analyzed in the per-protocol population; therefore, the potential effect of post-randomization exclusions on the preservation of the randomized comparison should be considered when interpreting the findings.

The actual inspired oxygen concentration was not measured in the LFNO group. Because FiO_2_ delivered through a low-flow nasal cannula varies with factors including minute ventilation, inspiratory flow, breathing pattern, and mouth breathing, the nominal FiO_2_ in the two treatment groups cannot be assumed to have been identical.

In addition, EtCO_2_ was measured by oral side stream capnography. Although the sampling site was separate from the nasal oxygen-delivery interface, HFNO may have influenced both the sampled gas and CO_2_ accumulation beneath the surgical drapes. Therefore, the lower measured EtCO_2_ observed with HFNO should be interpreted with consideration of these measurement-related effects. Chance baseline imbalance in measured EtCO_2_ was present between treatment groups within some ASA strata, particularly ASA III, and should be considered when interpreting the stratum-specific EtCO_2_ findings despite baseline adjustment.

Finally, physiological variables such as work of breathing, tidal volume, and transpulmonary pressure were not measured directly; therefore, the physiological mechanisms underlying the observed treatment-associated differences could not be determined.

Because intra-procedural bradypnea and CSB episodes were documented by an attending anesthesiologist who was aware of treatment allocation, the possibility of detection bias related to unblinded event ascertainment cannot be completely excluded, despite the use of predefined physiological criteria.

Strengths include the randomized design, ASA-stratified allocation, standardized MMAS, protocol-defined oxygen-delivery settings, objective sedation monitoring, and comprehensive assessment of respiratory and oxygenation variables.

From a clinical perspective, the treatment-associated differences observed in this study were most pronounced in patients with ASA physical status III undergoing PPV under REA and MMAS. Future multicenter studies should determine whether individualized HFNO settings and patient-selection strategies can further optimize respiratory stability during procedural sedation and identify patients most likely to benefit from HFNO.

## 5. Conclusions

In this single-center randomized trial, the primary outcome showed no between-group difference between HFNO at 40 L·min^−1^ (set FiO_2_ 0.40) and LFNO at 5 L·min^−1^ during MMAS for PPV, as no hypoxemia occurred in either treatment group. Treatment-associated differences were observed in secondary and prespecified respiratory outcomes, including BF and measured EtCO_2_ peri-procedurally, particularly across ASA strata. In prespecified ASA-stratified analyses, these differences were most apparent in ASA III patients, with higher BF, fewer CSB episodes, and lower measured EtCO_2_ with HFNO.

## Figures and Tables

**Table 1:** Baseline demographic and procedural characteristics of the study groups.

Variable		ASA I			ASA II			ASA III		
		LFNO	HFNO	ASD	LFNO	HFNO	ASD	LFNO	HFNO	ASD
		*n* = 22	*n* = 21		*n* = 29	*n* = 28		*n* = 35	*n* = 29	
Demographic characteristics										
Age (y), mean (SD)		70 (13)	71 (12)	0.08	72 (13)	74 (9)	0.18	72 (11)	70 (10)	0.19
BMI (kg·m^−2^), mean (SD)		26 (4)	27 (4)	0.25	26 (2)	26 (3)	0.00	27 (5)	28 (4)	0.22
Sex, *n* (%)	Female	11 (50%)	10 (48%)	0.04	17 (59%)	15 (53%)	0.12	20 (57%)	16 (55%)	0.04
	Male	11 (50%)	11 (52%)		12 (41%)	13 (47%)		15 (43%)	13 (45%)	
Mallampati class, *n* (%)	I	13 (59%)	12 (57%)	0.04	9 (31%)	8 (28%)	0.14	8 (23%)	6 (21%)	0.05
	II	9 (41%)	9 (43%)		20 (69%)	20 (72%)		27 (77%)	23 (79%)	
Comorbidities, *n* (%)	Respiratory diseases	0 (0%)	0 (0%)	0.00	1 (4%)	1 (4%)	0.05	2 (6%)	1 (3%)	0.12
	Cardiovascular disease	0 (0%)	0 (0%)	0.00	12 (41%)	11 (39%)	0.05	13 (37%)	10 (34%)	0.07
	Respiratory and cardiovascular diseases	0 (0%)	0 (0%)	0.00	3 (10%)	4 (14%)	0.12	5 (14%)	5 (17%)	0.08
	Other comorbidities	0 (0%)	0 (0%)	0.00	13 (44%)	12 (43%)	0.03	15 (43%)	13 (45%)	0.04
Characteristics of pars plana vitrectomy (PPV)										
	Lens luxation	3 (14%)	2 (10%)	0.13	2 (7%)	1 (4%)	0.13	2 (6%)	2 (7%)	0.02
	Epiretinal membrane	10 (45%)	8 (38%)	0.14	9 (31%)	9 (32%)	0.02	13 (37%)	9 (31%)	0.13
	Retinal detachment	5 (23%)	8 (38%)	0.33	15 (52%)	15 (54%)	0.03	15 (43%)	15 (52%)	0.18
	Vitreous hemorrhage	4 (18%)	3 (14%)	0.10	3 (10%)	3 (11%)	0.03	5 (14%)	3 (10%)	0.12
Oculocardiac reflex, *n* (%)		2 (9%)	3 (14%)	0.15	2 (7%)	1 (4%)	0.13	2 (6%)	3 (10%)	0.14
Duration of PPV (min), mean (SD)		65 (11)	63 (17)	0.09	65 (18)	68 (14)	0.08	60 (17)	63 (15)	0.18
Characteristics of analgosedation (MMAS)										
Oral premedication, *n* (%)										
midazolam (0.1 mg·kg^−1^; maximum 7.5 mg; age < 60 years)		8 (36%)	8 (36%)	0.04	10 (34%)	12 (43%)	0.03	7 (20%)	8 (28%)	0.18
diazepam (0.05 mg·kg^−1^; maximum 5.0 mg; age ≥ 60 years)		14 (64%)	13 (62%)		19 (66%)	16 (57%)		28 (80%)	21 (72%)	
Regional eye block, *n* (%)	Sub-Tenon block	12 (55%)	10 (48%)	0.14	14 (48%)	16 (57%)	0.18	18 (51%)	14 (48%)	0.06
	Retrobulbar block	10 (45%)	11 (52%)		15 (52%)	12 (43%)		17 (49%)	15 (52%)	
Time-averaged peri-procedural remifentanil infusion rate (ng·kg^−1^·min^−1^), mean (SD)		73 (12)	70 (18)	0.19	76 (17)	77 (23)	0.05	33 (11)	36 (13)	0.12
Duration of MMAS (min), mean (SD)		66 (15)	63 (17)	0.18	67 (17)	70 (14)	0.19	63 (13)	65 (12)	0.16
Duration of oxygenation (min), mean (SD)		77 (22)	75 (17)	0.10	77 (27)	80 (20)	0.13	75 (30)	76 (29)	0.03
Baseline physiological variables (T0)										
	SpO_2_ (%), mean (SD)	98 (2.0)	98 (1.9)	0.00	97 (2.1)	98 (2.0)	0.49	94 (2.6)	93 (1.7)	0.43
	BF (breaths·min^−1^), mean (SD)	15 (3.3)	13 (2.4)	0.69	15 (3.1)	14 (3.8)	0.29	14 (3.6)	14 (2.8)	0.00
	Measured EtCO_2_ (mmHg), mean (SD)	30 (5.7)	36 (3.9)	1.27	33 (3.7)	35 (6.9)	0.26	38 (3.3)	44 (3.3)	1.91
Recovery characteristics										
Pain score (NRS), mean (SD)		0.7 (0.4)	0.6 (0.5)	0.22	0.5 (0.4)	0.6 (0.5)	0.22	0.6 (0.5)	0.7 (0.5)	0.20
Aldrete score, mean (SD)		9.6 (0.5)	9.7 (0.5)	0.20	9.5 (0.5)	9.6 (0.5)	0.20	9.1 (0.5)	9.2 (0.4)	0.19
Time from oxygenation discontinuation to an Aldrete score of 9–10 (min), mean (SD)		6 (3.8)	5 (3.6)	0.27	5 (3.1)	5 (1.7)	0.00	5 (3.6)	6 (3.8)	0.27

Data are presented as mean (SD) or *n* (%). ASD, absolute standardized difference; ASA, American Society of Anesthesiologists physical status; BMI, body mass index; HFNO, high-flow nasal oxygenation; LFNO, low-flow nasal oxygenation; MMAS, moderate multimodal analgosedation; NRS, numeric rating scale (0–10 points); PPV, pars plana vitrectomy.

**Table 2:** Sedation assessment during moderate multimodal analgosedation according to oxygenation strategy and ASA physical status.

	ASA I		ASA II		ASA III	
	LFNO	HFNO	LFNO	HFNO	LFNO	HFNO
	(*n* = 22)	(*n* = 21)	(*n* = 29)	(*n* = 28)	(*n* = 35)	(*n* = 29)
PSI, mean ± SD						
T0	97.7 ± 2.0	97.8 ± 1.9	97.2 ± 2.1	97.8 ± 2.0	93.9 ± 2.6	93.2 ± 1.7
T1	85.6 ± 1.6	85.8 ± 1.7	85.5 ± 1.7	85.0 ± 1.6	81.1 ± 2.3	80.9 ± 2.1
T2	97.4 ± 1.9	96.8 ± 2.2	96.8 ± 1.9	97.6 ± 2.1	94.5 ± 2.0	94.6 ± 1.6
RSS, *n* (%)						
T0—RSS 2	17 (77%)	17 (81%)	26 (90%)	26 (93%)	29 (83%)	21 (72%)
T0—RSS 3	5 (23%)	4 (19%)	3 (10%)	2 (7%)	6 (17%)	8 (28%)
T1—RSS 2	16 (73%)	15 (71%)	14 (48%)	12 (43%)	11 (31%)	8 (28%)
T1—RSS 3	6 (27%)	6 (29%)	15 (52%)	16 (57%)	24 (69%)	21 (72%)
T2—RSS 2	15 (68%)	13 (62%)	23 (79%)	21 (75%)	27 (77%)	21 (72%)
T2—RSS 3	7 (32%)	8 (38%)	6 (21%)	7 (25%)	8 (23%)	8 (28%)

Data are presented as mean ± SD or *n* (%), as appropriate. Changes in PSI from T0 to T1 were compared between ASA III and ASA I–II patients within each oxygenation group; no significant differences were observed (LFNO, *p* = 0.126; HFNO, *p* = 0.716). At T1, the distribution of RSS 2 versus RSS 3 did not differ significantly between LFNO and HFNO in ASA III patients (*p* = 0.789). Data are presented for the per-protocol population (*n* = 164). ASA, American Society of Anesthesiologists physical status; HFNO, high-flow nasal oxygenation; LFNO, low-flow nasal oxygenation; PSI, Patient State Index; RSS, Ramsay Sedation Scale; SD, standard deviation; T0, pre-procedural; T1, peri-procedural; T2, post-procedural. RSS 2 indicates that the patient is cooperative, oriented, and tranquil; RSS 3 indicates that the patient responds to commands.

**Table 3:** Adverse events during moderate multimodal analgosedation (MMAS) for pars plana vitrectomy (PPV).

Variable	ASA I			ASA II			ASA III		
	LFNO	HFNO	*p*	LFNO	HFNO	*p*	LFNO	HFNO	*p*
	*n* = 22	*n* = 21		*n* = 29	*n* = 28		*n* = 35	*n* = 29	
Deeper-than-target sedation (PSI < 80)	0 (0%)	0 (0%)	–	1 (3%)	1 (4%)	–	4 (11%)	3 (10%)	–
Snoring	0 (0%)	0 (0%)	–	1 (3%)	0 (0%)	–	2 (6%)	0 (0%)	0.497
Upper airway obstruction	0 (0%)	0 (0%)	–	0 (0%)	0 (0%)	–	1 (3%)	0 (0%)	–
Apnoea	0 (0%)	0 (0%)	–	0 (0%)	0 (0%)	–	0 (0%)	0 (0%)	–
Nausea or vomiting	0 (0%)	0 (0%)	–	0 (0%)	1 (4%)	0.491	1 (3%)	0 (0%)	–
Involuntary movement or restlessness	0 (0%)	0 (0%)	–	1 (3%)	0 (0%)	–	0 (0%)	1 (3%)	0.453
Patient discomfort during PPV	0 (0%)	0 (0%)	–	1 (3%)	0 (0%)	–	1 (3%)	0 (0%)	–
Temporary surgical interruption	0 (0%)	0 (0%)	–	1 (3%)	0 (0%)	–	1 (3%)	0 (0%)	–

Data are presented as *n* (%). Comparisons between LFNO and HFNO within each ASA physical-status stratum were performed using the two-sided Fisher exact test because of sparse event counts. ASA, American Society of Anesthesiologists physical status; HFNO, high-flow nasal oxygenation; LFNO, low-flow nasal oxygenation; MMAS, moderate multimodal analgosedation; PPV, pars plana vitrectomy; PSI, Patient State Index.

## Data Availability

The de-identified data supporting the findings of this study may be available from the corresponding author upon reasonable request, subject to approval by the institution and applicable ethical and data-protection requirements.

## Abbreviations

The following abbreviations are used in this manuscript:

ASA	American Society of Anesthesiologists physical status
BF	Breathing frequency
CSB	Clinically significant bradypnea
HFNO	High-flow nasal oxygenation
LFNO	Low-flow nasal oxygenation
MMAS	Moderate multimodal analgosedation
PPV	Pars plana vitrectomy
PSI	Patient State Index
REA	Regional eye anesthesia

## References

[1] Hinkelbein J., Lamperti M., Akeson J., Santos J., Costa J., De Robertis E., Longrois D., Novak-Jankovic V., Petrini F., Struys M.M.R.F. (2018). European Society of Anaesthesiology and European Board of Anaesthesiology guidelines for procedural sedation and analgesia in adults. Eur. J. Anaesthesiol..

[2] Kumar C.M., Seet E., Chua A.W.Y. (2023). Updates in ophthalmic anaesthesia in adults. BJA Educ..

[3] Cheng S.-Y., Wu Y.-F., Gu X.-W., Zhao Q., Meng L.-H., Chen Y.-X., Zhao X.-Y. (2023). The incidence, risk factors, management and prognosis of postoperative systemic complications after ophthalmic surgery: A retrospective study at a tertiary, academic referral hospital over a decade. Ann. Med..

[4] Pucchio A., Pur D.R., Dhawan A., Sodhi S.K., Pereira A., Choudhry N. (2023). Anesthesia for ophthalmic surgery: An educational review. Int. Ophthalmol..

[5] McCusker S., Chaudhary V., Blew P., Mak M., Barbosa J., Qian J., Shanthanna H., Ligori T. (2020). Sedation during vitreoretinal surgery: Practice patterns in Canada. Can. J. Anaesth..

[6] Verschakelen T., Dewinter G. (2025). An overview of procedural sedation guidelines and their differences. Best Pract. Res. Clin. Anaesthesiol..

[7] van den Bosch O.F.C., Alvarez-Jimenez R., de Grooth H.J., Girbes A.R.J., Loer S.A. (2021). Breathing variability—Implications for anaesthesiology and intensive care. Crit. Care.

[8] Nay M.-A., Fromont L., Eugene A., Marcueyz J.-L., Mfam W.-S., Baert O., Remerand F., Ravry C., Auvet A., Boulain T. (2021). High-flow nasal oxygenation or standard oxygenation for gastrointestinal endoscopy with sedation in patients at risk of hypoxaemia: A multicentre randomised controlled trial (ODEPHI trial). Br. J. Anaesth..

[9] Barends C.R.M., Absalom A.R., Struys M.M.R.F. (2018). Drug selection for ambulatory procedural sedation. Curr. Opin. Anaesthesiol..

[10] Sneyd J. (2022). Developments in procedural sedation for adults. BJA Educ..

[11] O’Driscoll B.R., Howard L.S., Earis J., Mak V. (2017). British Thoracic Society guideline for oxygen use in adults in healthcare and emergency settings. BMJ Open Respir. Res..

[12] Leone M., Einav S., Chiumello D., Constantin J.-M., De Robertis E., Gama De Abreu M., Gregoretti C., Jaber S., Maggiore S.M., Pelosi P. (2020). Noninvasive respiratory support in the hypoxaemic peri-operative/periprocedural patient: A joint ESA/ESICM guideline. Intensive Care Med..

[13] Cortegiani A., Accurso G., Mercadante S., Giarratano A., Gregoretti C. (2018). High flow nasal therapy in perioperative medicine: From operating room to general ward. BMC Anesthesiol..

[14] Wyatt K.D., Goel N.N., Whittle J.S. (2022). Recent advances in the use of high flow nasal oxygen therapies. Front. Med..

[15] Al-Husinat L., Jouryyeh B., Rawashdeh A., Alenaizat A., Abushehab M., Amir M.W., Al Modanat Z., Battaglini D., Cinnella G. (2023). High-flow oxygen therapy in the perioperative setting and procedural sedation: A review of current evidence. J. Clin. Med..

[16] Genecand L., Agoritsas T., Ehrensperger C., Kharat A., Marti C. (2022). High-flow nasal oxygen in acute hypoxemic respiratory failure: A narrative review of the evidence before and after the COVID-19 pandemic. Front. Med..

[17] Lodeserto F.J., Lettich T.M., Rezaie S.R. (2018). High-flow nasal cannula: Mechanisms of action and adult and pediatric indications. Cureus.

[18] Sawase H., Ozawa E., Yano H., Ichinomiya T., Yano R., Miyaaki H., Komatsu N., Ayuse T., Kurata S., Sato S. (2023). Respiratory support with nasal high flow without supplemental oxygen in patients undergoing endoscopic retrograde cholangiopancreatography under moderate sedation: A prospective, randomized, single-center clinical trial. BMC Anesthesiol..

[19] Horiuchi A., Graham D.Y. (2020). High-flow nasal cannula oxygen therapy obscures severe respiration depression, providing an illusion of safety. Gastrointest. Endosc..

[20] Douglas N., Ng I., Nazeem F., Lee K., Mezzavia P., Krieser R., Steinfort D., Irving L., Segal R. (2018). A randomised controlled trial comparing high-flow nasal oxygen with standard management for conscious sedation during bronchoscopy. Anaesthesia.

[21] Modi P., Cates T.D. (2026). Home Oxygen Therapy. StatPearls.

[22] O’Reilly Nugent A., Kelly P.T., Stanton J., Swanney M.P., Graham B., Beckert L. (2014). Measurement of oxygen concentration delivered via nasal cannulae by tracheal sampling. Respirology.

[23] Ramsay M.A.E., Savege T.M., Simpson B.R.J., Goodwin R. (1974). Controlled sedation with alphaxalone-alphadolone. Br. Med. J..

[24] Miyake K., Higuchi H., Miyake S., Nishioka Y., Fujimoto M., Kurita E., Kawase A., Wakasugi Y., Miyawaki T. (2023). Evaluation of Sedation Levels Using SedLine during Intravenous Sedation for Dental Procedures: A Case-Series Study. Anesth. Prog..

[25] Aldrete J.A. (1995). The post-anesthesia recovery score revisited. J. Clin. Anesth..

[26] Mishima G., Sanuki T., Revie J., Pinkham M., Watanabe T., Kurata S., Tatkov S., Ayuse T. (2020). Nasal high flow improves ventilation during propofol sedation: A randomized cross-over study in healthy volunteers. Respir. Physiol. Neurobiol..

[27] Thiruvenkatarajan V., Sekhar V., Wong D.T., Currie J., Van Wijk R., Ludbrook G.L. (2023). Effect of high-flow nasal oxygen on hypoxaemia during procedural sedation: A systematic review and meta-analysis. Anaesthesia.

[28] Wang S., Ji J., Xiong C., Zhong W., Li L., Gong S., Lu J., Hei Z., Yao W., Chen C. (2024). Comparing oxygen therapies for hypoxemia prevention during gastrointestinal endoscopy under procedural sedation: A systematic review and network meta-analysis. J. Clin. Anesth..

[29] Campanella A., Guglielmi R., Zattera L., Jacas A., Ferrando C. (2026). High-flow nasal oxygen in the perioperative setting: A narrative review. Anaesth. Crit. Care Pain Med..

[30] Bradley W.P.L., Anderson J., Balkin M., Brewster D.J., Kayak E., Shariffuddin I.I., Tan P.C.F., Wong P. (2026). High-flow nasal oxygen in contemporary airway management: Enhancing physiological safety in anaesthesia—A narrative review. Healthcare.

[31] Thiruvenkatarajan V., Dharmalingam A., Arenas G., Wahba M., Liu W.-M., Zaw Y., Steiner R., Tran A., Currie J. (2022). Effect of high-flow vs. low-flow nasal plus mouthguard oxygen therapy on hypoxaemia during sedation: A multicentre randomised controlled trial. Anaesthesia.

[32] Yin X., Xu W., Zhang J., Wang M., Chen Z., Liu S., Xu Y., Xu S., Ji D., Wang J. (2024). High-flow nasal oxygen versus conventional nasal cannula in preventing hypoxemia in elderly patients undergoing gastroscopy with sedation: A randomized controlled trial. Int. J. Med. Sci..

[33] Lee M.-J., Cha B., Park J.-S., Kim J.S., Cho S.Y., Han J.-H., Park M.H., Yang C., Jeong S. (2022). Impact of high-flow nasal cannula oxygenation on the prevention of hypoxia during endoscopic retrograde cholangiopancreatography in elderly patients: A randomized clinical trial. Dig. Dis. Sci..

[34] Gu W.-J., Wang H.-T., Huang J., Pei J.-P., Nishiyama K., Abe M., Zhao Z.-M., Zhang C.-D. (2022). High flow nasal oxygen versus conventional oxygen therapy in gastrointestinal endoscopy with conscious sedation: Systematic review and meta-analysis with trial sequential analysis. Dig. Endosc..

[35] Lin Y., Zhang X., Li L., Wei M., Zhao B., Wang X., Pan Z., Tian J., Yu W., Su D. (2019). High-flow nasal cannula oxygen therapy and hypoxia during gastroscopy with propofol sedation: A randomized multicenter clinical trial. Gastrointest. Endosc..

[36] Zhang W., Yin H., Xu Y., Fang Z., Wang W., Zhang C., Shi H., Wang X. (2022). The effect of varying inhaled oxygen concentrations of high-flow nasal cannula oxygen therapy during gastroscopy with propofol sedation in elderly patients: A randomized controlled study. BMC Anesthesiol..

[37] Arakawa H., Kaise M., Sumiyama K., Saito S., Suzuki T., Tajiri H. (2013). Does pulse oximetry accurately monitor a patient’s ventilation during sedated endoscopy under oxygen supplementation?. Singap. Med. J..

[38] Askar H., Misch J., Chen Z., Chadha S., Wang H.-L. (2020). Capnography monitoring in procedural intravenous sedation: A systematic review and meta-analysis. Clin. Oral Investig..

